# Identification and characterization of CCAAT/Enhancer Binding proteindelta (C/EBPdelta) target genes in G_0 _growth arrested mammary epithelial cells

**DOI:** 10.1186/1471-2199-9-83

**Published:** 2008-10-01

**Authors:** Yingjie Zhang, Tong Liu, Pearlly Yan, Tim Huang, Jim DeWille

**Affiliations:** 1 Department of Veterinary Biosciences, Ohio State University, 1925 Coffey Road, Columbus, OH 43210, USA; 2 Department of Molecular Virology, Immunology and Medical Genetics, Ohio State University, 1645 Neil Avenue, Columbus, OH 43210, USA; 3 Molecular Biology and Cancer Genetics Program, Ohio State University, Comprehensive Cancer Center, Columbus, OH, USA

## Abstract

**Background:**

CCAAT/Enhancer Binding Proteinδ (C/EBPδ) is a member of the highly conserved C/EBP family of leucine zipper (bZIP) proteins. C/EBPδ is highly expressed in G_0 _growth arrested mammary epithelial cells (MECs) and "loss of function" alterations in C/EBPδ have been associated with impaired contact inhibition, increased genomic instability and increased cell migration. Reduced C/EBPδ expression has also been reported in breast cancer and acute myeloid leukemia (AML). C/EBPδ functions as a transcriptional activator, however, only a limited number of C/EBPδ target genes have been reported. As a result, the role of C/EBPδ in growth control and the potential mechanisms by which "loss of function" alterations in C/EBPδ contribute to tumorigenesis are poorly understood. The goals of the present study were to identify C/EBPδ target genes using Chromatin Immunoprecipitation coupled with a CpG Island (HCG12K) Array gene chip ("ChIP-chip") assay and to assess the expression and potential functional roles of C/EBPδ target genes in growth control.

**Results:**

ChIP-chip assays identified ~100 C/EBPδ target gene loci which were classified by gene ontology (GO) into cell adhesion, cell cycle regulation, apoptosis, signal transduction, intermediary metabolism, gene transcription, DNA repair and solute transport categories. Conventional ChIP assays validated the ChIP-chip results and demonstrated that 14/14 C/EBPδ target loci were bound by C/EBPδ in G_0 _growth arrested MCF-12A MECs. Gene-specific RT-PCR analysis also demonstrated C/EBPδ-inducible expression of 14/14 C/EBPδ target genes in G_0 _growth arrested MCF-12A MECs. Finally, expression of endogenous C/EBPδ and selected C/EBPδ target genes was also demonstrated in contact-inhibited G_0 _growth arrested nontransformed human MCF-10A MECs and in mouse HC11 MECs. The results demonstrate consistent activation and downstream function of C/EBPδ in growth arrested human and murine MECs.

**Conclusion:**

C/EBPδ target genes were identified by a global gene array approach and classified into functional categories that are consistent with biological contexts in which C/EBPδ is induced, such as contact-mediated G_0 _growth arrest, apoptosis, metabolism and inflammation. The identification and validation of C/EBPδ target genes provides new insights into the mechanistic role of C/EBPδ in mammary epithelial cell biology and sheds new light on the potential impact of "loss of function" alterations in C/EBPδ in tumorigenesis.

## Background

CCAAT/Enhancer Binding Proteinδ (C/EBPδ) is a member of the highly conserved C/EBP family of leucine zipper DNA binding proteins [1-3]. Evidence accumulated since their discovery in the late 1980's indicates C/EBP function in the transcriptional control of genes that function in cell growth, survival, differentiation, inflammation and apoptosis [1-3]. C/EBPδ gene expression is increased in human and mouse mammary epithelial cells in response to growth arrest induction by serum and growth factor withdrawal, contact inhibition and IL-6 family cytokine treatment [4-11]. Ectopic C/EBPδ expression induces growth arrest of mouse mammary epithelial and human chronic myelogenous leukemia cell lines [5,12]. Conversely, reducing C/EBPδ gene expression is associated with delayed growth arrest, genomic instability, impaired contact inhibition, increased cell migration and reduced serum dependence [5,13]. Consistent with a role as a candidate tumor suppressor gene, "loss of function" alterations in C/EBPδ gene expression have been reported in primary human breast cancer and acute myeloid leukemia (AML) [11,14-18]. In vivo experimental studies indicate that C/EBPδ plays a complex role in mammary epithelial cell fate determining programs as C/EBPδ is transiently induced in the mammary gland during the early "reversible" phase of mammary gland involution and C/EBPδ knockout female mice exhibit mammary gland ductal hyperplasia [19-22].

Studies focusing on the regulation of C/EBPδ have reported that C/EBPδ is regulated at the transcriptional, post-transcriptional and post-translational levels [6,23-25]. These findings demonstrate that the content and function of C/EBPδ is tightly controlled at multiple levels. The goal of the present study was to gain new insights into the functional role of C/EBPδ in mammary epithelial cell growth arrest by identifying C/EBPδ downstream target genes using a global gene array approach. The results identified candidate C/EBPδ target genes that were classified by gene ontology (GO) and functional annotation clustering into DNA binding, transcriptional regulation, cell adhesion, cell cycle regulation, apoptosis, signal transduction, intermediary metabolism, DNA repair and transport. These findings provide new insights into the broad range of functions impacted by C/EBPδ in mammary epithelial cell biology and suggest new mechanisms by which alterations in C/EBPδ could contribute to defects in growth control, differentiation and tumorigenesis.

## Results

### C/EBPδ is induced in growth arrested human mammary epithelial cells

**Figure 1:**
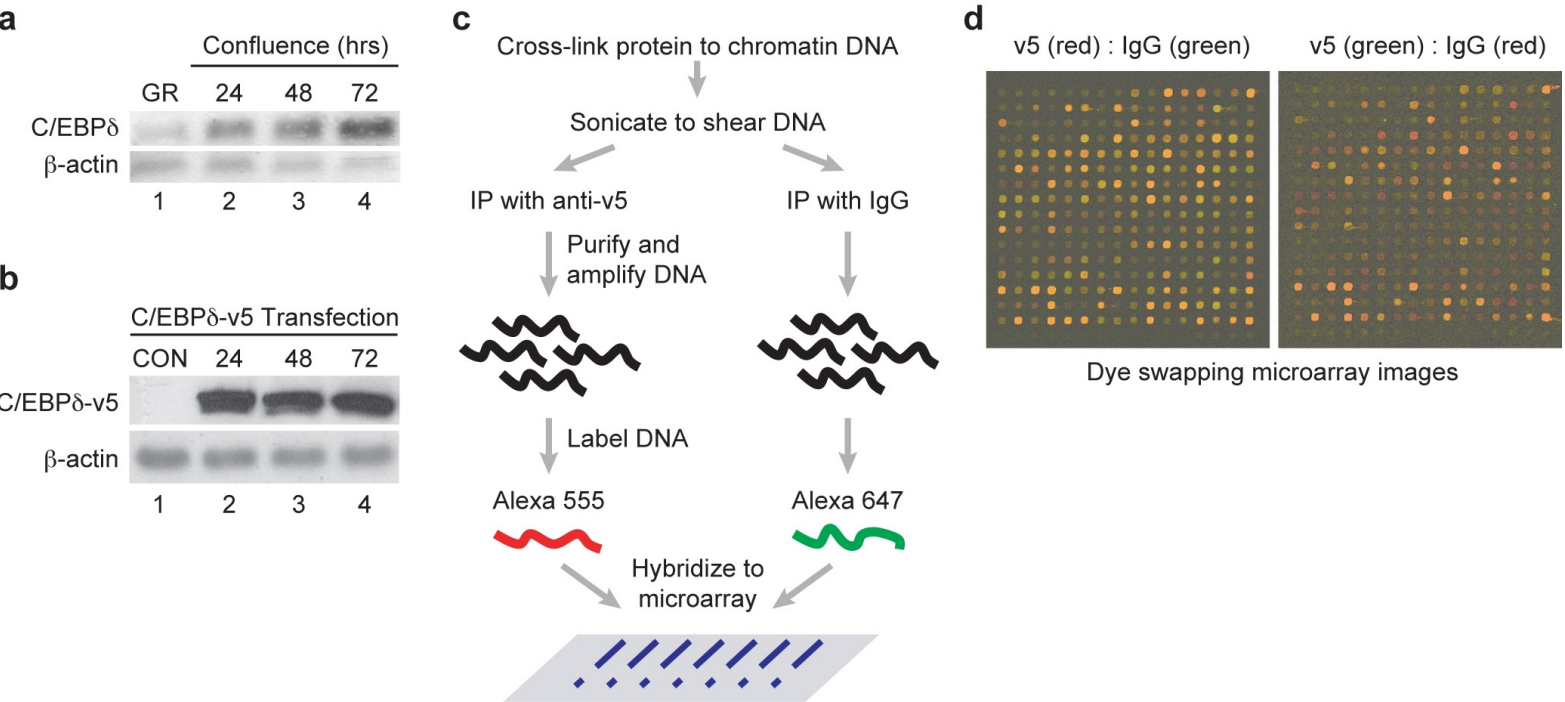
**C/EBPδ expression and C/EBPδ genomic target gene identification by ChIP-chip assay in MCF-12A cells**. a. C/EBPδ protein levels in whole cell lysates from growing and confluent, contact-inhibited MCF-12A cells. Lanes: (1) Exponentially growing; (2) Confluence (contact inhibition) induced growth arrest (24 hours); (3) 48 hours; (4) 72 hours. b. C/EBPδ-v5 protein levels in transfected MCF-12A cells. Lanes: (1) MCF-12A cells transfected with pcDNA3 vector (CON, control), (2) Confluent 24 hours, (3) Confluent 48 hour, (4) Confluent 72 hours. c. Schematic overview of the ChIP-chip protocol. d. HCG12K Array probed with ChIP isolated DNA coupled with Alexa 555 or Alexa 647 dyes. The data presented were derived from dye swapping experiments performed on the same microarray.

To identify C/EBPδ target genes we used the ChIP-chip assay, a technique that couples chromatin immunoprecipitation (ChIP) with (CpG) Island (CGI) microarray chip hybridization [26,27]. In the initial experiment, we validated the increase in C/EBPδ protein levels in MCF-12A human mammary epithelial cells growth arrested by contact inhibition for 24, 48 and 72 hours (Fig. 1a). We next transfected MCF-12A human mammary epithelial cells with a C/EBPδ-v5 fusion construct and demonstrated that the C/EBPδ-v5 protein was present at 24, 48 and 72 hours in contact inhibited MCF-12A cells, paralleling the results from experiments with endogenous C/EBPδ protein levels (Fig. 1b and Fig. 1a). Because available commercial and laboratory produced anti-C/EBPδ antibodies were not suitable for chromatin immunoprecipitation reactions the ChIP-chip assays were performed in contact-inhibited MCF-12A cells transfected with the C/EBPδ-v5 construct and the antibody interaction step was performed with a high affinity anti-v5 antibody. A schematic overview of the ChIP-chip protocol and representative microarray data images are presented (Fig. 1cd).

### Identification of and functional categories of C/EBPδ target genes

**Figure 2:**
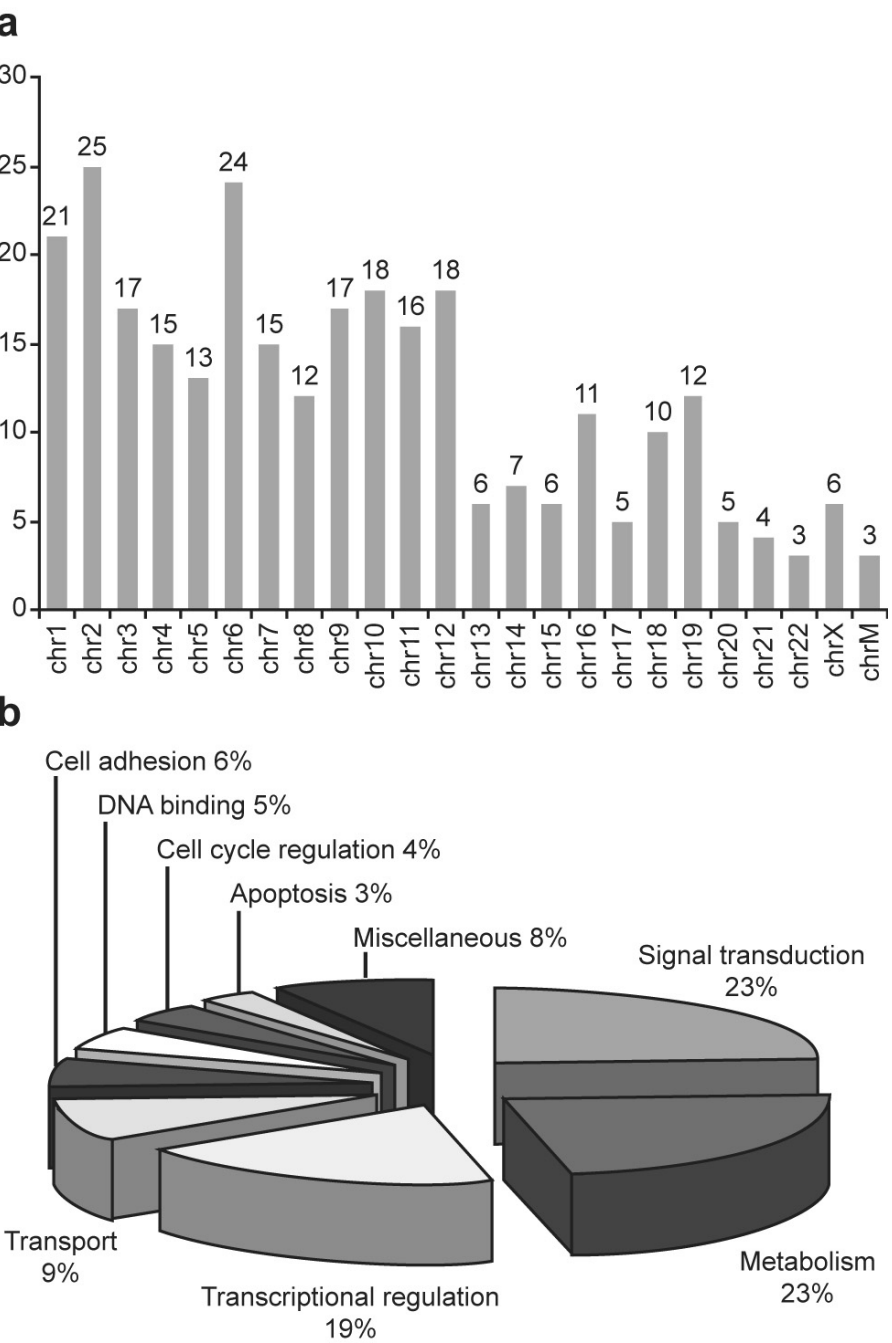
**Chromosomal localization and functional categories of C/EBPδ target genes**. A. Chromosomal localization of C/EBPδ-v5 bound candidate genomic targets identified by the C/EBPδ-v5 ChIP-chip assays using the UNH HCG12K array. b. C/EBPδ target genes were verified in authentic gene promoter regions and assigned to Functional Categories using the Database for Annotation, Visualization and Integrated Discovery (DAVID). The list of genes assigned to each category is presented in Table 1.

ChIP-chip results identified 289 candidate genomic regions from the UNH HCG12K array using a 2 fold enrichment threshold (C/EBPδ-v5 vs IgG control). Of these 289 genomic regions, 99 were identified in defined gene promoter regions (Table 1). C/EBPδ target genes are located on all human chromosomes, suggesting a broad and relatively unbiased distribution across the human genome (Fig. 2a). C/EBPδ target genes were identified and assigned to functional categories (Functional Annotation Clustering) using the Database for Annotation, Visualization and Integrated Discovery (DAVID) Bioinformatics Resource. C/EBPδ target gene functional categories include: signal transduction, metabolism, transcriptional regulation, cell adhesion, DNA binding, cell cycle control, apoptosis, and solute/metabolite transport (Fig 2b).

**Table 1:** C/EBPδ Target gene functional categories

**Signal transduction**		
**Gene Name**	**Gene Description**	**Gene ID**

ADM	Adrenomedullin	133

BAI3	brain-specific angiogenesis inhibitor 3	577

DTNA	dystrobrevin, alpha	1837

DVL3	dishevelled, dsh homolog 3 (Drosophila)	1857

EDG1	endothelial differentiation, sphingolipid G-protein-coupled receptor, 1	1901

GNG10	guanine nucleotide binding protein (G protein), gamma 10	2790

IRAK2	interleukin-1 receptor-associated kinase 2	3656

LOX	lysyl oxidase	4015

NPAS1	neuronal PAS domain protein 1	4861

CCL25	chemokine (C-C motif) ligand 25	6370

CDC42BPA	CDC42 binding protein kinase alpha (DMPK-like)	8476

INTS6	integrator complex subunit 6	26512

GTPBP2	GTP binding protein 2	54676

EPS15L2	epidermal growth factor receptor pathway substrate 15-like 2	55380

VAC14	Vac14 homolog (S. cerevisiae)	55697

ERBB2IP	erbb2 interacting protein	55914

ROBO3	roundabout, axon guidance receptor, homolog 3 (Drosophila)	64221

C9orf89	chromosome 9 open reading frame 89	84270

SPSB3	splA/ryanodine receptor domain and SOCS box containing 3	90864

HSP90AA1	heat shock protein 90 kDa alpha (cytosolic), class A member 1	3320

FGF9	fibroblast growth factor 9 (glia-activating factor)	2254

SCAP2	src family associated phosphoprotein 2	8935

GPR160	G protein-coupled receptor 160	26996

VDR	vitamin D (1,25- dihydroxyvitamin D3) receptor	7421

**Metabolism**		

OXA1L	oxidase (cytochrome c) assembly 1-like	5018

RPP30	ribonuclease P/MRP 30 kDa subunit	10556

THBS4	thrombospondin 4	7060

CKAP1	cytoskeleton associated protein 1	1155

DLD	dihydrolipoamide dehydrogenase	1738

ESD	esterase D/formylglutathione hydrolase	2098

LRP1	low density lipoprotein-related protein 1 (alpha-2-macroglobulin receptor)	4035

PSMB1	proteasome (prosome, macropain) subunit, beta type, 1	5689

RPL29	ribosomal protein L29	6159

MTMR6	myotubularin related protein 6	9107

ADAMTS5	ADAM metallopeptidase with thrombospondin type 1 motif, 5	11096

GCAT	lycine C-acetyltransferase (2-amino-3-ketobutyrate coenzyme A ligase)	23464

ADAT1	adenosine deaminase, tRNA-specific 1	23536

FLRT2	fibronectin leucine rich transmembrane protein 2	23768

MRPL35	mitochondrial ribosomal protein L35	51318

OTUB1	OTU domain, ubiquitin aldehyde binding 1	55611

USP48	ubiquitin specific peptidase 48	84196

ACBD5	acyl-Coenzyme A binding domain containing 5	91452

C9orf103	chromosome 9 open reading frame 103	414328

GANC	glucosidase, alpha; neutral C	2595

BCAT2	branched chain aminotransferase 2, mitochondrial	587

CRLF3	cytokine receptor-like factor 3	51379

ADPRH	ADP-ribosylarginine hydrolase	141

**Transcriptional regulation**		

KLF6	Kruppel-like factor 6	1316

DBP	D site of albumin promoter (albumin D-box) binding protein	1628

FLI1	Friend leukemia virus integration 1	2313

MEF2B	MADS box transcription enhancer factor 2, polypeptide B	4207

POLR2F	polymerase (RNA) II (DNA directed) polypeptide F	5435

POU2F1	POU domain, class 2, transcription factor 1	5451

SOX4	SRY (sex determining region Y)-box 4	6659

TBP	TATA box binding protein	6908

ZNF20	zinc finger protein 20	7568

CSDA	cold shock domain protein A	8531

RFXANK	regulatory factor X-associated ankyrin-containing protein	8625

TAF1A	TATA box binding protein (TBP)-associated factor, RNA polymerase I, A	9015

SSBP2	single-stranded DNA binding protein 2	23635

MKL2	MKL/myocardin-like 2	57496

TGIF2	TGFB-induced factor 2 (TALE family homeobox)	60436

IRX6	iroquois homeobox protein 6	79190

ESX1	extraembryonic, spermatogenesis, homeobox 1 homolog (mouse)	80712

ZNF573	zinc finger protein 573	126231

ALX4	aristaless-like homeobox 4	60529

**Transporters**		

KCND2	potassium voltage-gated channel, Shal-related subfamily, member 2	3751

PCM1	pericentriolar material 1	5108

TUSC3	tumor suppressor candidate 3	7991

SLC25A14	solute carrier family 25 (mitochondrial carrier, brain), member 14	9016

HGS	hepatocyte growth factor-regulated tyrosine kinase substrate	9146

HCN4	hyperpolarization activated cyclic nucleotide-gated potassium channel 4	10021

SLC40A1	solute carrier family 40 (iron-regulated transporter), member 1	30061

MCART1	mitochondrial carrier triple repeat 1	92014

CCBE1	collagen and calcium binding EGF domains 1	147372

**Cell cycle regulation**		

SEPT7	septin 7	989

RCC1	regulator of chromosome condensation 1	1104

PAPD5	PAP associated domain containing 5	64282

DIRAS3	DIRAS family, GTP-binding RAS-like 3	9077

**DNA binding**		

TOP2B	topoisomerase (DNA) II beta 180 kDa	7155

HIST1H4F	histone 1, H4f	8361

KCMF1	potassium channel modulatory factor 1	56888

XPC	xeroderma pigmentosum, complementation group C	7508

MSH5	mutS homolog 5 (E. coli)	4439

**Cell Adhesion**		

GP5	glycoprotein V (platelet)	2814

ITGB8	integrin, beta 8	3696

PCDH9	Protocadherin 9	5101

RSHL1	radial spokehead-like 1	81492

THBS4	thrombospondin 4	7060

**Apoptosis**		

TIA1	cytotoxic granule-associated RNA binding protein	7072

BCL2L1	BCL2-like 1	598

RNF34	ring finger protein 34	80196

**Miscellaneous**		

HSPCA	heat shock protein 90 kDa alpha (cytosolic), class A member 1	3320

OTOF	otoferlin	9381

LOH12CR1	loss of heterozygosity, 12, chromosomal region 1	118426

MYEOV2	myeloma overexpressed 2	150678

TMEM87A	transmembrane protein 87A	25963

MTPN	myotrophin	136319

DISC1	disrupted in schizophrenia 1	27185

### Chromatin immunoprecipitation (ChIP) and RT-analysis of C/EBPδ target genes

**Figure 3:**
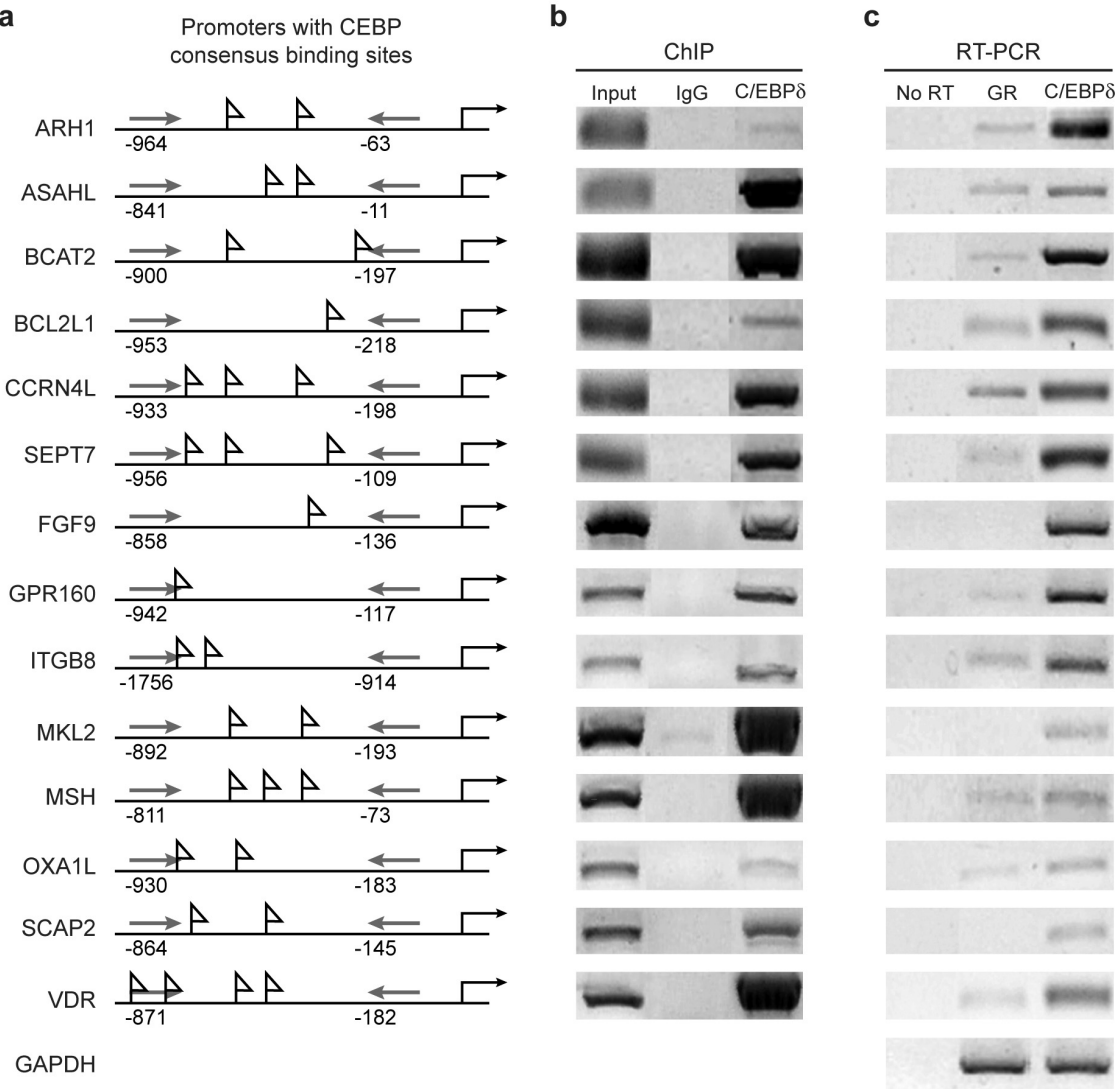
**Conventional ChIP and RT-PCR analysis of selected C/EBPδ ChIP-chip target genes**. a. C/EBPδ ChIP-chip target gene promoters. C/EBPδ target gene promoters are shown with gene-specific primers (→) and computer predicted C/EBP consensus sites (▶) Gene-specific human primer pairs are presented in Table 2. b. Conventional ChIP assays. Whole cell lysates were isolated from MCF-12A cells transfected with pCDNA3.1-hC/EBPδ-v5 and growth arrested by contact inhibition. Conventional ChIP assays performed with anti-v5 and IgG (negative control) antibodies. Input lane is derived from direct PCR amplification of genomic DNA. c. C/EBPδ target gene expression: RT-PCR analysis. Total RNA was isolated from MCF-12A cells transfected with pCDNA3.1-hC/EBPδ-v5 and cultured under exponentially growing (GR) or contact inhibition conditions. Total RNA was reverse transcribed and PCR amplified using gene-specific primers. No RT = PCR amplification of RNA samples without RT. GAPDH was used as a non-C/EBPδ inducible RNA expression control.

We next used conventional chromatin immunoprecipitation (ChIP) assays to confirm the interaction between C/EBPδ and selected candidate gene promoters in MCF-12A mammary epithelial cells. MCF-12A cells were transfected with the C/EBPδ-v5 construct, growth arrested by contact inhibition and conventional ChIP assays performed on 14 C/EBPδ candidate genes from diverse functional categories with proximal promoters containing at least one consensus C/EBP binding site (Fig. 3a). ChIP assay results were positive for 14/14 C/EBPδ candidate target gene promoters tested, although the degree of positive detection varied across the 14 target genes (Fig. 3b).

**Table 2:** Forward (F-) and reverse (R-) primers for ChIP and RT-PCR assays (human)

**Gene name**	**Primers for ChIP**	**Primers for RT-PCR**
DIRAS3	F- ctcacaggcaagggagaaag;	F- ccgaagggccaagtggaggaagc;
	R- tacaggttggggaggaactg	R- tggtgaggcagccccgttgtt

ASAHL	F- gcagagacacaccagcagag;	F- gtggctcaagactccagagg;
	R- gtaagccgtggaggaggag	R- tgcttcgaagttttccgact

BCAT2	F- aagaggccttgtgaggtcaa;	F- ccgctgaatggtgttatcct;
	R- ctcgctggaaagagctgagt	R- tctccttcagctccttctgg

BCL2L1	F- agagctcttgcgtctggaag,	F- agagctcttgcgtctggaag;
	R- ggacttctcaatggggttca	R- ggacttctcaatggggttca

CCRN4L	F- cctgaccatgtctttgctca;	F- ctggagcccattgatcctaa;
	R- cgcaggcggtctaaaataag	R- ggtaggccaggatttcttcc

SEPT7	F- ggagtgtgagctccaagagg;	F- aatagttgataccccaggat;
	R- cttgcttacgcacgctacag	R- gagcaatgaagtataaacaacac

FGF9	F- ctctcgcagtgcatctttca;	F- tgagaagggggagctgtatgga;
	R- tcccatccgaccgtaataag	R- gtgaatttctggtgccgtttagtc

GPR160	F- aaggttgcccgtctctgac;	F- gctctcgcttcgtcctacac;
	R- gcctcggaaaacaaatagcc	R- taggggctggtttgtttgac

ITGB8	F- caagtcctcacacccatcct;	F- gctctcgcttcgtcctacac;
	R- ccttcccagtaaacggaaca	R- taggggctggtttgtttgac

MKL2	F- ctctgtcctgtgtgccattc;	F- ctgtcctccccacaaacact;
	R- cgtgactgggaagggttaaa	R- gatctgcagttgcaggaaca

MSH5	F- atgttcaccgctttgagtcc;	F- gagacgctgctgatgtacca;
	R- ccagcctagagatccgacag	R- cctgatgagttgggtccagt

OXA1L	F- agcctcccaaagtgatgaga;	F- agaatgatgcccctgataacctt;
	R- gtcgcgattgtcctctgatt	R- gacgcgtcatttcagcatttttc

SCAP2	F- cgagctcagaggccatcgtagggt;	F- ctcccaaagatgctgaaga;
	R- gaagatcttcccggccccagaaga	R- tgcttgttagtggattgcttat

VDR	F- ctggatgattttgtgagca;	F- cagtttgggaggtcgaggta;
	R- aattttcatcgaccgtcgtc	R- gaatgagagtgggggtctga

ChIP-chip and direct ChIP assays address in situ protein/DNA binding but do not determine if DNA binding results in increased expression of the downstream target gene. To investigate the relationship between C/EBPδ promoter binding and C/EBPδ target gene expression MCF-12A cells were transfected with the C/EBPδ-v5 construct, growth arrested by contact inhibition and total RNA isolated for RT-PCR analysis. The RT-PCR results demonstrated that mRNA levels of 14/14 of the selected C/EBPδ target genes are significantly induced in MCF-12A cells transiently transfected with the C/EBPδ-v5 construct under contact inhibition, growth arrest conditions (Fig. 3c). The degree of C/EBPδ target gene expression as assessed by mRNA content was variable, possibly reflecting the complex nature of individual target gene transcriptional activation as well as individual target gene mRNA stability. Taken together, the conventional ChIP and RT-PCR results verified that the ChIP-chip assays identified authentic C/EBPδ target genes.

### C/EBPδ and C/EBPδ target genes are induced in confluent (contact inhibited) human and mouse mammary epithelial cell lines

**Figure 4:**
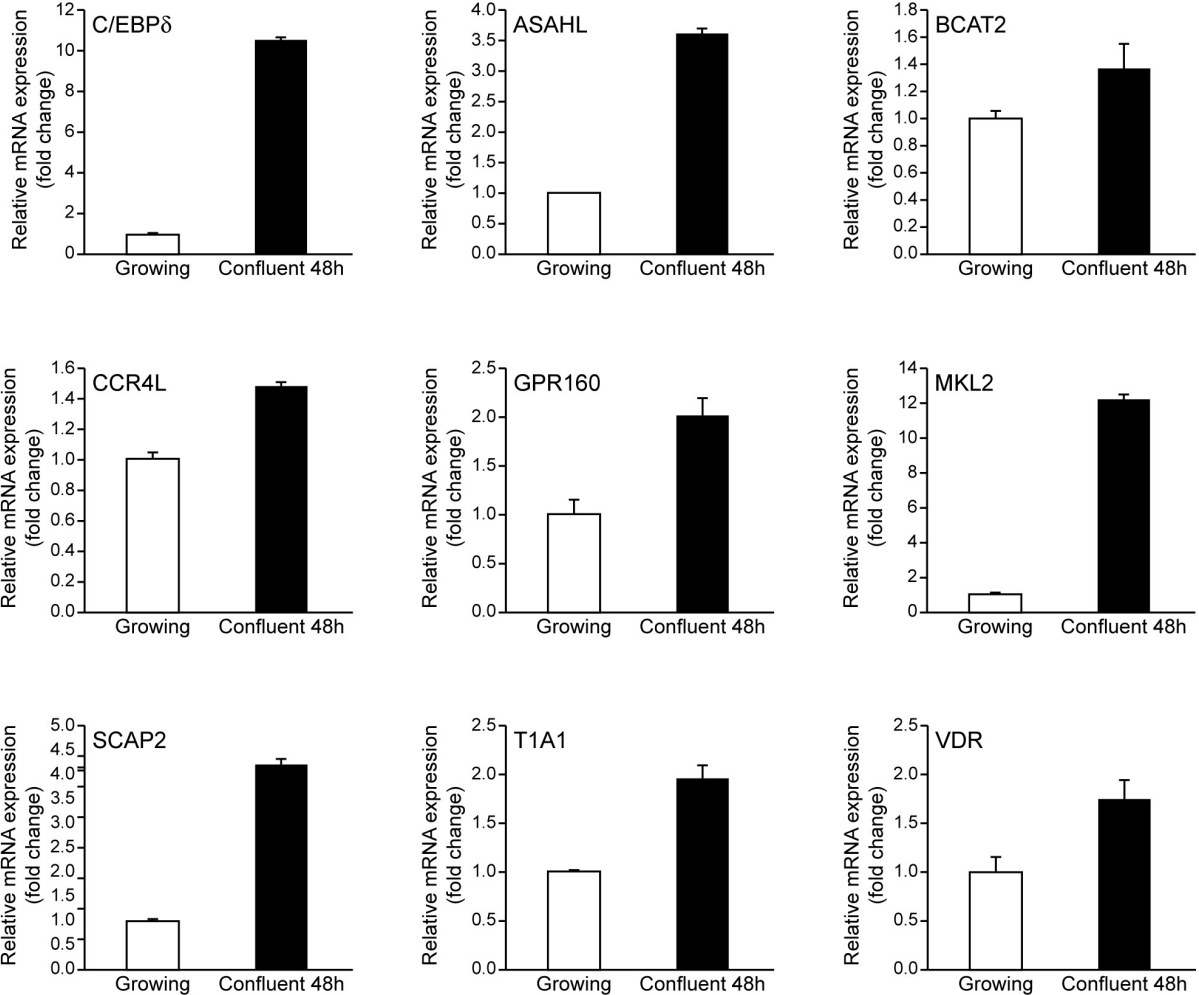
**C/EBPδ and C/EBPδ target gene mRNA levels are expressed in confluent, G_0 _growth arrested human mammary epithelial cells**. Growing MCF-10A cells were maintained at ~50% confluence in CGM; confluent MCF-10A cells were grown to confluence and maintained in CGM for 48 hours. Real Time PCR analysis was performed using the LightCycler 480 Real Time PCR System. The gene specific human primers pairs are presented in Table 3.

In previous work we reported that C/EBPδ expression is highly induced in growth arrested and IL-6 cytokine treated primary human mammary epithelial cells, MCF-12A and MCF-10A mammary epithelial cell lines [9]. To extend these findings in the current study we assessed the expression of C/EBPδ and selected C/EBPδ target genes in 48 hour confluent, G_0 _growth arrested MCF-10A mammary epithelial cells. The results demonstrated that G_0 _growth arrest was associated with an approximately 10-fold induction of C/EBPδ mRNA compared to exponentially growing MCF-10A cells (Fig. 4). Consistent with the growth arrest induction of C/EBPδ, the mRNA levels of selected C/EBPδ target genes were also induced, with fold induction of C/EBPδ target genes varying from ~.5–12 fold induction (Fig. 4).

**Table 3:** C/EBPδ and C/EBPδ target gene primers (mouse)

		Sequence (5'-3')	Length (bp)^a^	Accession No.^b^
C/EBPδ	FW	CGACTTCAGCGCCTACATTGA	171 bp	NM007679
		
	RV	CTAGCGACAGACCCCACAC		

ASAHL	FW	GTCCTCCTGACTTCCTGG	225 bp	NM025972
		
	RV	CCTGCCACTAAGCCTCAC		

BCAT2	FW	ATGAAGGCAAGCAACTCC	227 bp	NM009737
		
	RV	TGGACAGACCTTTCCCTATT		

GP5	FW	CGCCAGCCTGTCGTTCT	185 bp	NM008148
		
	RV	GCCTGTTATTGGGACTTTCAC		

ITGB8	FW	TTCTCCTGTCCCTATCTCCA	302 bp	NM177290
		
	RV	TGAGACAGAT TGTGAGGGTG		

MKL2	FW	CTGTGGTCGTCAAGCAAGA	398 bp	NM153588
		
	RV	TGTGTTTGGTGCCGAGTTT		

MSH5	FW	CGACTCCTGAGCCACATC	295 bp	NM013600
		
	RV	TGGCATCTATGTCAGGGTC		

OXA1L	FW	CGGTTCTATTGCCGTTGG	225 bp	NM026936
		
	RV	CACCCACTCCTCTTTCCTTT		

PCDH9	FW	ACAGCCACCACGGTCCTCTA	219 bp	NM001081377
		
	RV	CCCTTGTTGTTCCCGCTCAC		

SCAP2	FW	AGTGAAGATGGACGAGCAA	199 bp	NM018773
		
	RV	TCCTACCCACCAGCCATA		

T1A1	FW	GAGAAGGGCTATTCGTTTG	208 bp	NM009383
		
	RV	GTCCATACTGTTGTGGGTTT		

VDR	FW	CAACGCTATGACCTGTGAA	299 bp	NM009504
		
	RV	GCAGGATGGCGATAATGT		

XPC	FW	TCCTGGGAGATACCTTCG	337 bp	NM009531
		
	RV	AAAGAGCAGCAGGCAGTA		

GAPDH	FW	CTCACTGGCATGGCCTTCCG	293 bp	XM001473623
		
	RV	ACCACCCTGTTGCTGTAGCC		

Note. FW: forward primer; RV: reverse primer.

^a^Amplicon length in base pairs.

^b^Genbank accession number of corresponding gene, availabe at

**Figure 5:**
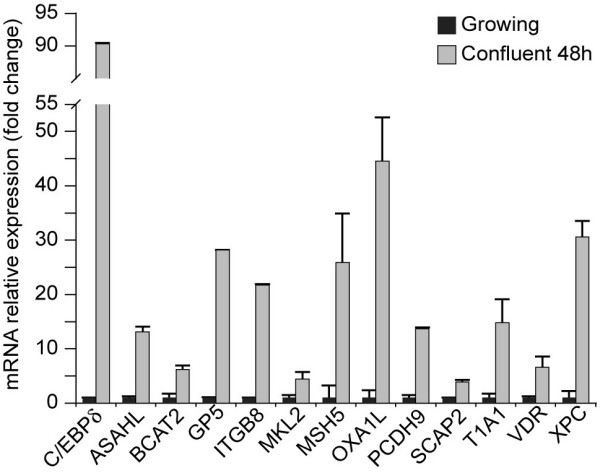
**C/EBPδ and C/EBPδ target gene mRNA levels are increased in confluent, G_0 _growth arrested mouse mammary epithelial cells**. Growing HC11 cells were maintained at ~50% confluence in CGM; confluent HC11 cells were grown to confluence and maintained in CGM for 48 hours. Real Time PCR analysis was performed using the LightCycler 480 Real Time PCR System. The gene specific primers are presented in Table 3. Real Time PCR data is normalized to the GAPDH control.

To extend the current results to mouse MECs we compared C/EBPδ and selected C/EBPδ target gene mRNA levels in growing and contact-inhibited, G_0 _growth arrested HC11 cells, a nontransformed mouse mammary epithelial cell line. The results confirmed the growth arrest induction of C/EBPδ and demonstrated parallel induction of selected C/EBPδ target gene mRNAs (Fig. 5a). The growth arrest inducible induction of C/EBPδ was dramatic (~90 fold), the growth arrest induction of selected C/EBPδ target genes varied from ~3–50 fold (Fig. 5). These results extend the association between C/EBPδ and the expression of C/EBPδ target genes to include both human and mouse derived nontransformed mammary epithelial cell lines.

## Discussion

This study identified C/EBPδ target genes using a "ChIP-chip" global gene array approach. The functional categories of a significant number of the C/EBPδ target genes are consistent with known biological responses associated with C/EBPδ expression and function. A significant number of studies have demonstrated that C/EBPδ gene expression is induced in contact-inhibited cells and the "ChIP-chip" analyses performed in this study identified C/EBPδ target genes that function in cell adhesion, a key aspect contact inhibition mediated growth arrest including, IGTB8, LOX, PCDH9, THBS4, and RSHL1 (Table 1) [28]. C/EBPδ induction of IGTB8 (Integrin B8) may be particularly relevant in breast cancer as IGTB8 inhibits epithelial cell growth by activating TGF-β [29,30]. In addition, LOX (lysyl oxidase), a cell-associated enzyme that functions in extracellular matrix biology has been identified as a tumor suppressor gene in gastric cancer [31]. However, the role of LOX is cancer biology is complex as LOX has also been shown to enhance breast cancer cell migration [32].

Additional C/EBPδ target genes function in the regulation of growth factor signaling, tumor suppression and transcription including: ERBB2IP, IRAK2, EDG1, INTS6, SCAP2, VDR, KLF6, MKL2, FLI1, TUSC3 and SOX4 (Table 1). ERBB2IP (Erbin) inhibits growth factor signaling by disrupting Sur-8/Ras/Raf complex formation interaction [33]. INTS6 (DICE1), a DEAD box protein that exhibits tumor suppressor activity, is hypermethylated and downregulated in prostate cancer [34]. VDR (vitamin D receptor), a member of the steroid hormone nuclear receptor superfamily, functions in calcium and noncalcium related cellular responses to vitamin D [35]. It is of interest that the VDR is required for vitamin D-induced growth arrest of breast and prostate derived cell lines and C/EBPδ is required for vitamin D-induced growth arrest of human breast (MCF-7) and prostate (LnCAP) cells [36,37]. These results indicate that C/EBPδ target genes play key roles in growth inhibitor signaling, cell-cell and cell matrix interactions and transcriptional regulation.

The C/EBPδ ChIP-chip results also identified three genes (BCL2L1, TIA-1, RNF34) that function in apoptosis. Reports from our lab and others demonstrate that C/EBPδ is expressed at the onset of mouse mammary gland involution [20,21,38]. It is of interest that BCL2L1 (*bcl-x*), a gene associated with pro- and anti-apoptotic functions was identified as a C/EBPδ target gene by the ChIP-chip assay. The primary BCL2L1 transcript can be alternatively spliced into two variants that encode proteins with opposing functions: Bcl-xL (anti-apoptotic) and Bcl-xS (pro-apoptotic) [39,40]. Bcl-xL is the most abundant Bcl-2 family member expressed in mammary epithelial cells and conditional deletion of the *bcl-x *gene from the mouse mammary epithelium enhances apoptosis during the initial phase of mammary gland involution [41]. Interestingly, Bcl-xS levels increase during mammary gland involution, resulting in a decrease in the Bcl-xL/Bcl-xS ratio in the involuting mammary gland [42]. A second apoptosis-related C/EBPδ target gene identified was TIA-1, an RNA binding protein that exhibits both pro- and anti-apoptotic activity [43,44]. These results suggest that C/EBPδ may function in the transcriptional control of BCL2L1 and TIA-1 but the pro- or anti-apoptotic functions are determined by posttranscriptional events. The third apoptosis-related C/EBPδ target gene identified in study is RNF34, an anti-apoptotic protein that is associated with activation of nuclear factor-κB (NF-κB) and increased levels of Bcl-xL [45]. In addition to the identification of growth control/tumor suppressor genes, the C/EBPδ ChIP-chip analysis identified eight inflammation related genes, including ADM, IRAK2, CCL25, OTUB1, KLF6, DBP, RFXANK and GP5 (Table 1). These findings are consistent with a well-established functional role of C/EBPδ in the acute phase response, inflammation and wound healing [23,46,47].

The ChIP-chip analysis also identified C/EBPδ target genes that encode proteins that function in general energy metabolism, including lipid metabolism, metabolite transport and mitochondrial energy-related functions (Table 1). These results are consistent with early reports documenting the key role of C/EBPδ in the 3T3-L1 fibroblast → adipocyte differentiation program [48,49].

The ChIP-chip analysis also identified a significant number of C/EBPδ target genes that function as transcriptional regulatory proteins. These results suggest that C/EBPδ initiates a biological response that is amplified by C/EBPδ target genes that also function as transcriptional regulatory proteins. Five C/EBPδ target genes are classified as homeobox genes (POU2F1, TGIF2, IRX6, ESX1L and ALX4) (Table 1). The potential role of C/EBPδ in the expression of homeobox genes suggests that C/EBPδ may influence cell fate or cell lineage determination. It has recently been shown that C/EBPδ inhibits growth and promotes self renewal of human limbic stem cells, suggesting a potential role for C/EBPδ in the maintenance of stem cell pluripotency [50]. These results suggest that C/EBPδ may play a previously unrecognized regulatory role in cell lineage determination in the mammary gland or possibly in mammary gland stem cell populations.

The ChIP-chip results identified C/EBPδ target genes that function specifically in neuronal differentiation and development (FGF9, MTPN, ROBO3, NPAS1, and DVL3) (Table 1). Early studies in our laboratory found that mouse brain expresses relatively high levels of C/EBPδ mRNA compared to other C/EBP family members [51]. In addition, the initial report that described the phenotype of C/EBPδ -/- mice reported selectively enhanced contextual fear conditioning, suggesting a role for C/EBPδ in learning or memory [52]. It is of interest that DTNA, a gene that functions in neuromuscular synaptic transmission was also identified as a C/EBPδ target gene and that C/EBPδ target genes were identified that function in differentiation and development of muscle cells and pattern formation of limb buds including MKL2, MEF2B and ALX4 [53]. These results suggest that epithelial cells may express a subset of genes that retain residual neural related or neuromuscular-related functions.

## Conclusion

This is the first report to utilize the ChIP-chip assay to identify C/EBPδ target genes. The new C/EBPδ target genes identified by the ChIP-chip analysis are associated with biological responses previously associated with C/EBPδ expression, such as growth arrest, cell adhesion, inflammation, energy metabolism and apoptosis. Gene expression analyses performed in human and mouse mammary epithelial cell lines confirm the link between the expression of C/EBPδ, C/EBδ target genes and the G_0 _growth arrest state. These results provide new insights into the functional role of C/EBPδ and C/EBPδ target genes in mammary epithelial cell growth control and suggest new avenues of investigation to define the role of C/EBPδ and C/EBPδ target genes in mammary tumorigenesis.

## Methods

### Cell culture and transient transfections

The immortalized, nontransformed MCF-12A and MCF-10A human mammary epithelial cell lines were obtained from American Type Culture Collection. MCF12A and MCF-10A cell lines were cultured in DMEM/F-12 phenol red free media (Invitrogen) supplemented with 5% horse serum, 20 ng/ml human recombinant EGF, 100 ng/ml cholera toxin, 10 μg/ml bovine insulin, 500 ng/ml hydrocortisone, 100 U/ml penicillin and 100 μg/ml streptomycin (Complete Growth media, CGM). Growth arrest was induced by culturing confluent MCF-12A or MCF-10A cells in CGM or switching near confluent cultures to media containing 0.5% horse serum plus antibiotics (Growth arrest media, GAM). MCF-12A cells were transiently transfected with 5 μg of a v5 tagged C/EBPδ expression construct (pCDNA3.1-hC/EBPδ-v5) using the Lipofectamine Plus transfection system (Invitrogen). Three hours later transfected cells were washed with 1× PBS, returned to CGM for 48 hours. All transfection experiments were performed in triplicate and repeated 2–3 times. HC11 cells (mouse immortalized mammary epithelial cell line) were grown in complete growth media (CGM) containing RPMI 1640 medium (Invitrogen) containing 10% FBS and supplemented with 10 ng/ml epidermal growth factor, 10 μg/ml insulin, 50 units/ml penicillin, 50 μg/ml streptomycin and 500 ng/ml fungizone in a humidified incubator at 37°C and 5% CO_2_. Exponentially growing HC11 cells were cultured at 30–50% confluence in CGM, confluent HC11 cells were grown to confluence and retained in CGM for 48 hours.

### Chromatin immunoprecipitation CpG island microarray ("ChIP-Chip") and ChIP assays

Isolation of C/EBPδ-associated genomic DNA was performed using the Chromatin Immunoprecipitation Assay Kit (Upstate) and following Upstate ChIP protocols. Anti-v5 epitope antibody (Invitrogen) (non-cross reactive with endogenous MCF-12A proteins) was used in the primary immunoprecipitation reaction. Mouse nonspecific IgG (Upstate) was used as a non-specific antibody control for the ChIP assays. Briefly, 5 × 10^6 ^MCF-12A cells were cross-linked with 1% formaldehyde (10 minutes, 37°C), washed 2× with PBS (4°C), pelleted by centrifugation and resuspended in 200 μl SDS lysis buffer supplemented with protease inhibitors. Cell lysates were sonicated to shear DNA to 0.5–2.0 kb in length (verified by agarose gel analysis). Sonicated lysates were centrifuged to remove debris, diluted 1:10 in dilution buffer and used for IP with 2 μg anti-v5 antibody or nonspecific mouse IgG control. After immunoprecipitation, pellets were washed with 1 ml Low Salt Immune Complex Wash Buffer, High Salt Immune Complex Wash Buffer and LiCl Immune Complex Wash Buffer and TE buffer. Bead precipitates were eluted twice with fresh elution buffer (1% SDS, 0.1 M NaHCO_3_) and eluates were pooled and heated at 65°C for 4 hours to reverse protein-DNA crosslinks. DNA was purified by phenol extraction and ethanol precipitation. To confirm C/EBPδ/target promoter binding, optimized, nested PCR was performed with 2.5 μl of the 50 μ1 DNA preparation plus promoter specific primers. Specific PCR products were assessed by agarose gel electrophoresis. An optimized two-step PCR amplification was then performed on the ChIP recovered DNA. The first amplification step involved a 3 cycle random primer amplification including: 8 μl ChIP DNA, 2 μ1 5× Sequenase Buffer, 1 μ1 of 40 μM primer A (5'-GTT TCC CAG TCA CGA TCN NNN NNN NN), 1.5 μ1 10 mM DNTP's, and 1 μ1 Sequenase (US Biochemical, Sequenase Kit Ver. 2.0) was incubated at 94°C for 2 min, 10°C for 5 min followed by 37°C for 8 min. The random primer incorporation reactions were then increased to a final volume of 60 l by the addition of 40 l of RNAse/DNAse-free water (Invitrogen). The second amplification step included 15 μ1 of the DNA product from step one, 8 μ1 MgCl_2_, 10 μ1 10× PCR Buffer, 2 μ1 50× aa-dUTP/dNTP's, 1 μ1 Primer B (5'-GTT TCC CAG TCA CGA TC 100 pm/μ1), 1 μ1 Taq polymerase (QIAGEN) plus 63 μ1 of RNAse/DNAse-free water. The following amplification/nucleotide incorporation program was used: 92°C for 30 s, 40°C for 30 s, 50°C for 30 s, 72°C for 1 min × 34 cycles. A confirmatory agarose gel was run with 5 μ1 of PCR product to visualize the DNA and confirm the size range of ~300–1000 bp in length.

PCR amplified anti-v5 and IgG ChIP isolated DNA was purified using the CyScribe GFX Purification Kit (Amersham, catalogue # 27-9602-02). DNA was resuspended and vortexed in vials containing Alexa 647 (green fluorescent) or Alexa 555 (red fluorescent) dye (Molecular Probes) in 2 μ1 100% DMSO (Sigma). Following complete dissolution of the dye 8 μ1 aa-dUTP was added and the sample was vortexed and incubated for 1 hour at room temperature in the dark. Following dye-coupling, samples were purified separately using the CyScribe GFX Purification Kit (Amersham) and the eluent volume reduced to 5 μ1 for hybridization by SpeedVac (45 min, medium heat setting). Hybridization of the labeled DNA sample to the UHN 12 k Human CpG Arrays was performed by the Ohio State University Comprehensive Cancer Microarray Core Laboratory. Briefly, CPG array slides were prehybridized in a solution containing 100 μ1 of DIG Easy Hyb solution (Roche), 5 μ1 of 10 mg/ml calf thymus DNA (Invitrogen) and 5 μ1 of 10 mg/m1 L yeast tRNA (Invitrogen) at 65°C for 2 min and then cooled to room temperature. The hybridization solution (85 μ1 total volume) containing the pooled Alexa 647 and Alexa 555 labeled DNA was mixed and incubated at 65°C for 2 min, cooled to room temperature and the pipetted onto the CPG array slides. A 24 × 60 mm glass coverslip (Corning) was placed over the hybridization droplet and the arrays was place into a hybridization chamber containing a small amount of DIG Easy Hyb solution in the bottom to maintain a humid environment. The arrays were incubated in a 37°C incubator for 18 hours. After hybridization, the slides were sequentially washed with 1× SSC and 0.1% SDS for 15 min in 50°C water bath, 1× SSC, and 0.1× SSC at room temperature. Slides were spun dry at 640 rpm for 15 min and the fluorescent signal scanned using a GenePix 4000B scanner. For each independent experiment the v5-antibody-ChIP DNA and the mouse IgG-ChIP control DNA fluro dye labeling was swapped to reduce the effect of dye bias on the microarray data. A 2 fold hybridization signal intensity (antiv5 ChIP vs the IgG ChIP) was used to identify C/EBPδ-v5 binding targets. Only those spots satisfying the 2 fold cut-off value in both of the two dye swapping microarray experiments were used for downstream bioinformatics analysis. Array spots with a size (diameter) less than 70% of the normal size or having a signal-to-noise ratio of less than 2.5 fold were eliminated from the analysis. We also determined that no reliable signal was produced from control spots containing *Arabidopsis *DNA. The conventional ChIP assays were performed by isolation of C/EBPδ-associated (C/EBPδ-v5) genomic DNA using the Chromatin Immunoprecipitation Assay Kit (Upstate) and following Upstate ChIP protocols.

### Bioinformatic and statistical analysis

CGI microarray gene information was obtained from the UHN Microarray Center's CpG Island Database . Genome sequences and annotations were obtained from the UCSC Genome Bioinformatics Site . All CGI hits were mapped to promoter, exonic, intronic, and intergenic regions according to the locations of RefSeq genes. Promoters were defined as 5 kb upstream to the annotated translation start sites. Statistical analysis was performed using Excel based software. Functional gene categories were identified and Functional Annotation Clustering performed using resources available at the Database for Annotation, Visualization and Integrated Discovery (DAVID) . Hypothetical genes and genes without GO assignments are not shown. The Alibaba2 program located at the BIOBASE gene regulation website  was used to identify potential C/EBP binding sites within the target promoters. Information about C/EBP family transcription factors was obtained from TRANSFAC 7.0-Public database in the BIOBASE website. Three independent experiments were performed.

### Reverse transcription -PCR (RT-PCR)

Total RNA was isolated using RNABee (TelTest, Inc.). One g RNA samples were treated with amplification grade DNase I and reverse transcribed with an oligo(dT) primer in 20 μ1 using the SuperScript First-Strand Synthesis System for RT-PCR from Invitrogen. One μ1 cDNA aliquots were amplified by gene specific primers. PCR amplification products were analyzed by agarose gel electrophoresis, and photographed using an Alpha Innotech Imagine System.

### mRNA isolation and Real Time PCR

Total mRNA was isolated using RNAzol B (Tel-Test, Inc., Friendswood, TX) according to the manufacturer's protocols. Total mRNA (1 μg) was reverse transcribed using the reverse transcriptase kit (Invitrogen, Carlsbad, CA). The reverse transcription products were amplified by Real-time PCR using the LightCycler^® ^480 Real-Time PCR System (Roche, Indianapolis, IN). Amplification was performed in a total volume of 20 μL containing 10 μL of a 2×SYBR Green PCR master mix, 0.2 μL of forward and reverse primers and 1 μL cDNA in each reaction. PCR specificity was verified by assessing the melting curves of each amplification product. Real-time PCR data were normalized to the glyceraldehyde-3-phosphate dehydrogenase (GAPDH) mRNA control. The primers used are presented in Table 2. The fold change in specific mRNA levels was calculated using the comparative CT (ΔΔCT) method. Results presented as mean ± SEM of the fold changes derived from three experiments with triplicate analyses performed for each treatment.

## Abbreviations

(C/EBPδ): CCAAT/Enhancer Binding Proteinδ; (ChIP-chip): chromatin immunoprecipitation-microarray chip; (MECs): mammary epithelial cells: UHN: University Health Network; HCG12K: Human CpG Island 12 K.

## Authors' contributions

YZ and JDW developed the experimental design. YZ performed cell culture, transfection, ChIP-chip, and RT-PCR luciferase assays. TL performed cell culture and Real-Time PCR assays. THMH pioneered the development of the ChIP-chip technology, provided CpG island arrays and extensive advice. PY provided technical advice in the design and implementation of the ChIP-chip assays, performed microarray hybridizations and assisted in the interpretation of the data. TJDW and YZ were the primary authors of the manuscript, however, all authors read and contributed to the final manuscript.
